# Supplementary material for: Assessment of Gastrointestinal Symptoms and Dyspnea in Patients Hospitalized due to COVID-19: Contribution to Clinical Course and Mortality
Source: J Clin Med. 2022 Mar 25;11(7):1821. doi: 10.3390/jcm11071821 (PMC8999487; doi:10.3390/jcm11071821)
Supplement: Supplementary file 1 [file jcm-11-01821-s001.zip › jcm-1635073-supplementary.pdf]

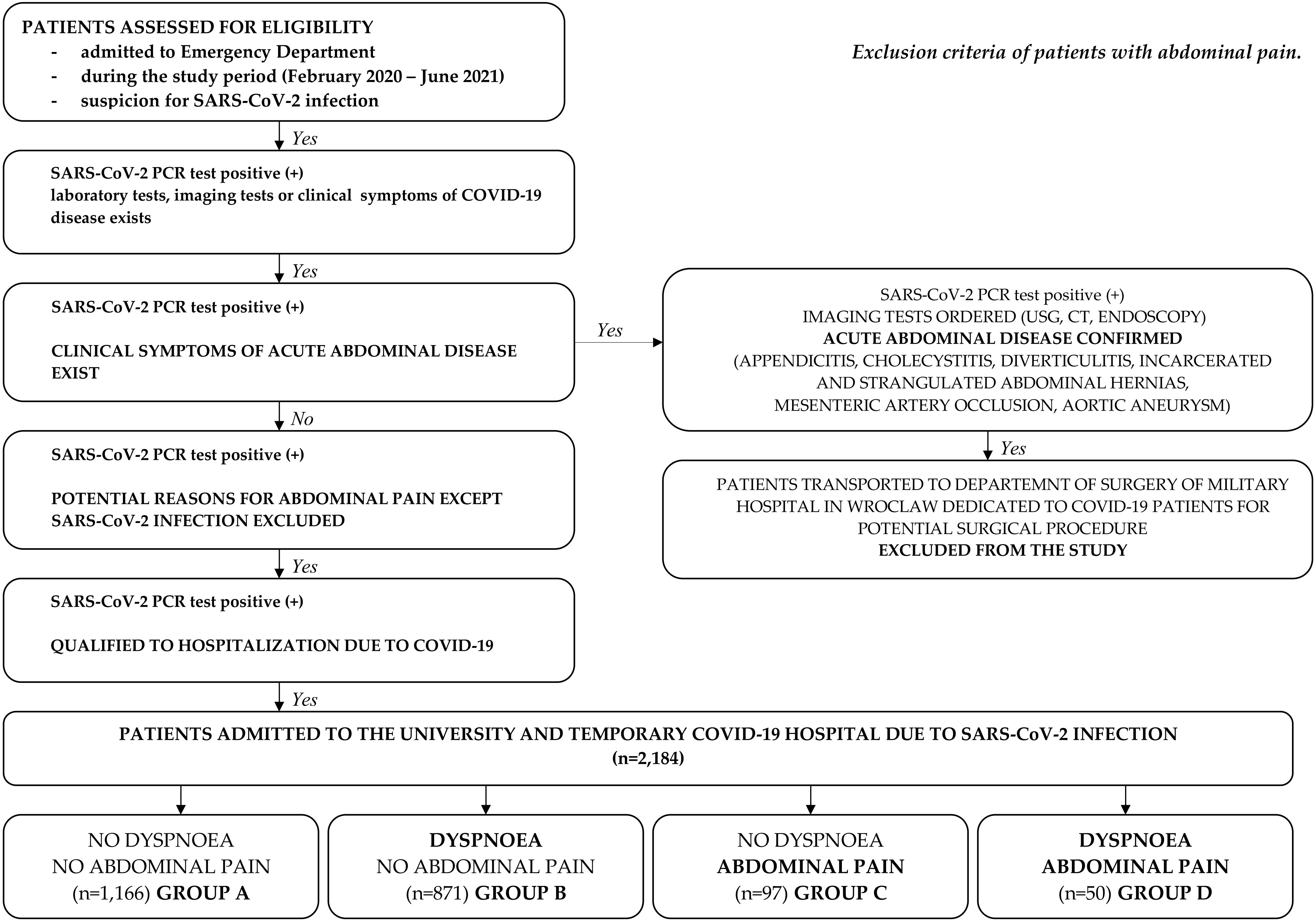

Exclusion criteria of patients with abdominal pain.
